# Hysterectomy rates per resident in final year of training in teaching hospitals: an ecologic study

**DOI:** 10.61622/rbgo/2025rbgo24

**Published:** 2025-04-30

**Authors:** Luiza Nestori Chiozzotto, Nino José Wilson Moterani, Laura Bresciani Bento Gonçalves Moterani, Vinicius César Moterani, Francisco José Candido dos Reis

**Affiliations:** 1 Faculdade de Medicina de Marília Marília SP Brazil Faculdade de Medicina de Marília, Marília, SP, Brazil.; 2 Faculdade de Medicina de Marília Departamento de Ginecologia e Obstetrícia Marília SP Brazil Faculdade de Medicina de Marília, Departamento de Ginecologia e Obstetrícia, Marília, SP, Brazil.; 3 Universidade de São Paulo Faculdade de Medicina de Ribeirão Preto Departamento de Ginecologia e Obstetrícia Ribeirão Preto SP Brazil Faculdade de Medicina de Ribeirão Preto, Universidade de São Paulo, Departamento de Ginecologia e Obstetrícia, Ribeirão Preto, SP, Brazil.; 4 Universidade Estadual Paulista Faculdade de Medicina de Botucatu Botucatu SP Brazil Faculdade de Medicina de Botucatu, Universidade Estadual Paulista, Botucatu, SP, Brazil.

**Keywords:** Hysterectomy, Medical staff, hospital, Hospitals, teaching, Learning curve, Education, medical, Clinical competence, Students, medical, Physicians, Surgical procedures, operative

## Abstract

**Objective:**

Analyze the hysterectomy rates per resident in graduation year in teaching hospitals in the state of São Paulo (Brazil).

**Methods:**

We selected teaching hospitals in the state of São Paulo and gathered information from two public databases to estimate the hysterectomy rates per resident in their final year of training between 2009 and 2019.

**Results:**

Between 2009 and 2019, there was a 37.5% increase in the number of residents in their final year of training, a 4.31% increase in the number of hysterectomies, and a drop in the hysterectomy rates per resident of 24.1%. The reduction of the rate of hysterectomy per resident was more pronounced for vaginal route (46.4%) followed by abdominal route (23.3%). The ratio of laparoscopic hysterectomy per resident increased 264% during the period, however, this route was used in only 7% of the surgeries in 2019.

**Conclusions:**

The hysterectomy rates per resident in their final year of training showed a notable reduction. This trend, particularly pronounced in vaginal and abdominal routes, signals a shift towards minimally invasive techniques.

## Introduction

Hysterectomy is the most commonly performed gynecological surgery in several countries, consisting of uterus removal for treatment of various gynecological diseases.^(1,2)^ The possible routes are abdominal, vaginal, and laparoscopic.^(1,3)^ Each has its advantages and disadvantages, with the choice depending on the diagnosis, health conditions and anatomy of the patient, hospital technical availability, and the surgeon's preference and experience.^(3)^ Although the vaginal and laparoscopic routes have less impact on the patient's recovery, the most used route is the abdominal.

The medical residency program in Obstetrics and Gynecology aims to specialize and capacitate physicians in clinical and surgical interventions related to women's health.^(4)^ Currently, the milestones designed by the Brazilian Federation of Obstetrics and Gynecology Associations (FEBRASGO) define that each resident must have acquired surgical competencies and that, by the end of the program, they will be able to perform hysterectomy through various surgical approaches.^(5)^

The resident physicians must participate in several surgeries throughout their training to acquire competencies and follow an adequate learning curve. Residents will practice these skills depending on availability and demand at their educational institution. We estimated that the number of residents in Brazil has increased; therefore, the number of hysterectomies performed per resident has decreased throughout the years. In this context, there is concern regarding the acquisition of this competence.

This study aimed to analyze the hysterectomy rates per resident in the last year of training within teaching hospitals located in the state of São Paulo (SP), Brazil.

## Methods

### Study design

We performed a retrospective and descriptive study of public databases, using information from the following databases: Certificate Consultation System of the National Medical Residency Commission (SisCNRM) and Hospital Information System of the Unified Health System (SIH/SUS).^(6,7)^ We obtained data on the annual number of resident doctors in their graduation year and the annual number of non-oncological hysterectomies in teaching hospitals in the state of SP from 2009 to 2019. This study follows the STROBE guideline.

### Ethics approval and consent to participate

Research that exclusively involves public domain data and does not identify research participants does not require ethical approval. We followed the Declaration of Helsinki.

### Data source and variables

We selected Obstetrics and Gynecology Medical Residency programs in cities with at least 200 thousand inhabitants, totaling 15 cities and 31 teaching hospitals (Chart 1).

**Chart 1:** Teaching hospitals and their respective cities

City	Teaching hospital
Botucatu	*Hospital das Clínicas da Faculdade de Medicina de Botucatu*
Bragança Paulista	*Hospital Universitário São Francisco na Providência de Deus*
Campinas	*Hospital das Clínicas da Universidade Estadual de Campinas*
	*Hospital Maternidade Celso Pierro (PUCCAMP)*
Carapicuíba	*Hospital Geral de Carapicuíba*
Catanduva	*Hospital Padre Albino (UNIFIPA)*
Jundiaí	*Hospital Universitário da Faculdade de Medicina de Jundiaí*
Limeira	*Santa Casa de Limeira*
Marília	*Hospital das Clínicas da Faculdade de Medicina de Marília* *Unidade Materno Infantil*
Presidente Prudente	*Hospital Domingos Leonardo Ceravolo*
Ribeirão Preto	*Hospital das Clínicas da Faculdade de Medicina de Ribeirão Preto*
	*Santa Casa de Ribeirão Preto*
Santos	*Hospital Guilherme Álvaro*
	*Santa Casa da Misericórdia de Santos*
São José do Rio Preto	*Hospital de Base da Faculdade de São José do Rio Preto*
São Paulo (metropolitan area)	*Conjunto Hospitalar Mandaqui*
	*Hospital Geral do Grajaú (UNISA)*
	*Hospital Santa Marcelina*
	*Hospital São Paulo da Escola Paulista de Medicina (UNIFESP)*
	*Hospital Ipiranga Unidade de Gestão Assistencial II*
	*Hospital Maternidade Leonor Mendes de Barros*
	*Hospital das Clínicas da Faculdade de Medicina da Universidade de São Paulo*
	*Hospital da Mulher Pérola Biyeton (Centro de Referência da Saúde da Mulher)*
	*Hospital Municipal e Maternidade Escola Dr. Mario de Moraes Altenfelder Silva*
	*Hospital Municipal Dr. Carmino Caricchio*
	*Santa Casa de Misericórdia de São Paulo*
	*Hospital do Servidor Público Municipal*
	*Hospital da Mulher Maria José dos Santos Stein de Santo André (FMABC)*
	*Hospital Municipal Dr. Mauro Pires Rocha de Campo Limpo*
Sorocaba	*Hospital Santa Lucinda (PUCSP)*
Taubaté	*Hospital Municipal Universitário de Taubaté*

SisCNRM is a database used to monitor medical residency programs. We extracted tables with the number of residents in their final year of training at teaching hospitals located in the state of São Paulo annually from 2009 to 2019.

The SIH/SUS contains information about hospital procedures performed in the Brazilian public health system (SUS). We extracted tables containing the number of non-oncological hysterectomies classified by route performed in the selected teaching hospitals annually from 2009 to 2019.

We calculated hysterectomy rates per resident in each city annually from 2009 to 2019 by dividing the number of surgeries by the number of residents in their final year of training.

We excluded data from *Hospital Ana Costa* (Santos) and Hospital do Servidor Público Francisco Morato Oliveira (city of São Paulo) due to lack of data on the number of hysterectomies.

### Statistical analysis

We aimed to identify if there is a variation in the proportion of hysterectomies per resident. To compare the hysterectomies per resident rate ratio on the first and last year of the study we used the Poisson rate ratio. An exact Poisson test was applied to compare the hysterectomies per resident rate ratio in 2009 and 2019. The same procedure was also done in hysterectomies per resident per route. We further generated a line and a heatmap graphs in the Displayr program (https://www.displayr.com/, accessed in February 2024).^(8)^

## Results

Table 1 shows the number of residents in their final year of training and hysterectomies in the selected teaching hospitals in the state of São Paulo between 2009 and 2019. In total, we identified 2,426 residents in their final year of training. There were 192 residents in 2009 and 264 in 2019, with an increase of 37.5%. In total, we counted 54,397 hysterectomies. We identified 4,825 hysterectomies performed in 2009 and 5,033 in 2019, with an increase of 4.31%. The most used route was abdominal (77.22%), followed by vaginal (19.14%) and laparoscopic (3.64%).

**Table 1:** Number of residents in their final year of training and of hysterectomies performed between 2009 and 2019 in the selected cities

Year	Residents in final year	Hysterectomies	Abdominal hysterectomies	Vaginal hysterectomies	Laparoscopic hysterectomies
2009	192	4,825	3,710	1,046	69
2010	197	4,916	3,718	1,123	75
2011	194	4,830	3,704	1,026	100
2012	197	4,565	3,486	961	118
2013	207	4,817	3,765	900	152
2014	215	5,052	3,886	1,000	166
2015	225	5,316	4,166	950	200
2016	223	5,079	3,896	969	214
2017	254	5,019	3,886	874	259
2018	258	4,945	3,876	790	279
2019	264	5,033	3,915	772	346
2009 - 2019	2426	54,397	42,008	10,411	1,978

**Figure 1:**
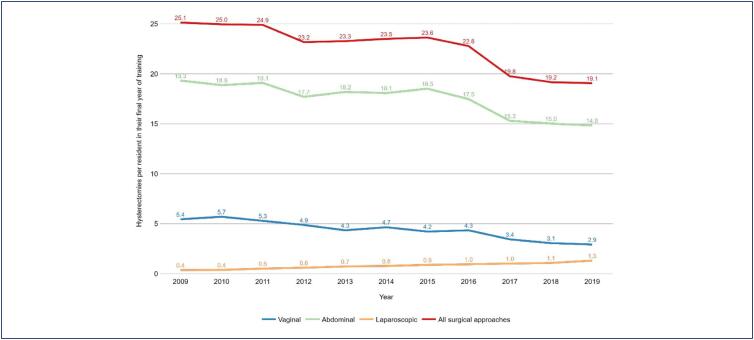
Hysterectomy rate per resident in their final year of training between 2009 and 2019

Figure 1 shows the trend in the rates of hysterectomies per resident in their final year between 2009 and 2019 in the state of SP. There has been a trend towards decreasing rates of hysterectomies per resident over the years, with the highest value of 25.1 in 2009 and the lowest value of 19.1 in 2019. The rates of abdominal and vaginal hysterectomies follow the same trend. Laparoscopic hysterectomies show an increasing trend, although they remain in low value.

When comparing the first and last year of the study, the rate ratio of total hysterectomies per resident was 0.759 (95% CI: 0.729 – 0.789, p <0,001), therefore a 24.1% reduction. The rate ratio of abdominal hysterectomies per resident was 0.767 (95% IC: 0.733 – 0.802, p < 0.001), therefore a 23.3% reduction. The rate ratio of vaginal hysterectomies per resident was 0.536 (95% IC: 0.488 - 0.589, p < 0.001), therefore a 46.4% reduction. The rate ratio of laparoscopic hysterectomies was 3.64 (95% IC: 2.809 - 4.793, p < 0.001), therefore a 3.64 times increase.

**Figure 2:**
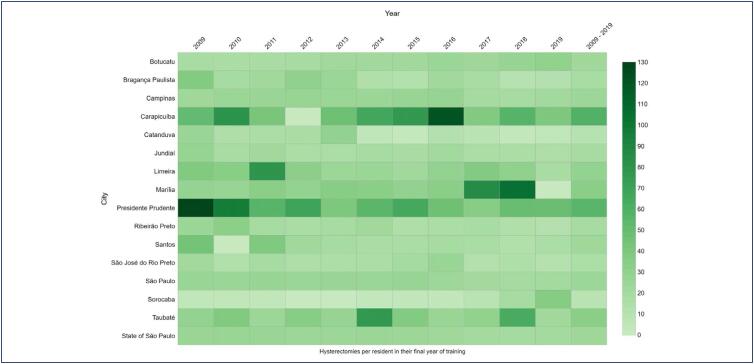
Heatmap of hysterectomy rates per resident in their final year of training in the cities of the selected hospitals between 2009 and 2019

Figure 2 shows hysterectomy rates per resident in final year between 2009 and 2019 for each selected city. In 2019, the city with the most hysterectomies per resident was Presidente Prudente (48.4), followed by Carapicuíba (39.3), Sorocaba (35.0) and Botucatu (29.4). On average, between 2009 and 2019, the city with the most hysterectomies per resident was Carapicuíba (59.0), followed by Presidente Prudente (55.3), Taubaté (32.3) and Marília (32.2).

## Discussion

Between 2009 and 2019, the number of residents in their final year of training had a noteworthy increase (37.5%), while the number of hysterectomies performed in the same period had a lower growth (4.31%) across the state of São Paulo. The hysterectomy rates per resident showed a consistent decline, attributed to the continual increase in resident numbers. This downward trend may potentially impact the training of resident physicians as they are exposed to fewer procedures, posing a challenge to the acquisition of essential surgical skills.

The latest Medical Demography in Brazil technical report highlighted that there is a large gap between the number of undergraduate medical students (1.05 students per 1,000 inhabitants in 2021) and the number of doctors under the specialization process (0.21 resident doctors per 1,000 inhabitants in 2021).^(9)^ As a result, the number of positions for medical residency in Brazil is annually increasing through policies that expand the medical residency programs, such as "Pró Residências". The Ministry of Education and Health implements these policies to equalize the number of recently graduated doctors and the number of positions for medical residency programs, in addition to trying to improve the service of medical specialties in the health system.^(10,11)^ In the Obstetrics and Gynecology specialization, for example, there was an increase of approximately 80.3% in residency positions between 2010 and 2019.^(12)^ Increasing the number of residency positions is essential to meet the demand for doctors seeking specialization and providing specialized care.^(10,11)^ However, this large number of new residents could harm the learning curve of resident physicians if the number of hysterectomies performed starts to decrease for each trainee.

Currently, the number of hysterectomies in Brazil tends to decrease when looking at data from the Oswaldo Cruz Foundation.^(13)^ In this research, specifically in the selected institutions, the number of hysterectomies between 2009 and 2019 remained relatively stable in the state of São Paulo. The trend toward decreasing the number of hysterectomies performed may occur due to better clinical control of diseases that require hysterectomy.^(14,15)^ In other words, physicians and patients possibly chose clinical treatments over hysterectomy.^(14,15)^ Therefore, new technologies allow the evolution of clinical treatments though, may lead to fewer opportunities for residents to perform surgeries.

Studies performed in the United States of America evaluated resident and program director confidence in hysterectomy training, they concluded that most residents feel confident to perform hysterectomies through different routes by graduation.^(16,17)^ Another American study determined that graduating residents might be underprepared to perform hysterectomies according to the perception of fellowship program directors.^(18)^ In Japan, a study sought to estimate an ideal number of abdominal and vaginal hysterectomy surgeries per resident to satisfactorily acquire these skills, mentioning that it is necessary 75 abdominal surgeries in three years and more than 25 vaginal surgeries in the same period.^(19)^ An editorial from the American College of Obstetrics and Gynecology evaluates the downward trend in hysterectomy rates and mentions that each resident needs 30 to 60 procedures throughout training years to become competent.^(2)^ We found no Brazilian study that explores and indicates the number of hysterectomies performed by residents to evaluate the learning curve. A Brazilian study analyzed the epidemiology of the practice of hysterectomies in the city of São Paulo between 2008 and 2018, which indicated a drop in the number of hysterectomies performed.^(14)^ Another Brazilian study mentions a 16% reduction in hysterectomies performed in the country between 2008 and 2017 and the need for better assessment of residents to understand whether the learning curve is being improved.^(20)^ However, the study did not calculate the hysterectomy rates per resident for analysis.

This study benefits from the analysis of a substantial volume of hysterectomies within a clearly delineated setting. However, inherent limitations of ecological studies, including potential selection bias and the inability to analyze confounding variables, should be acknowledged. The number of residents in final year of training was used as a reference, as the analysis included periods of one year. Residents may perform hysterectomies in institutions that are not the head hospital of their residency programs, and outside the public health system, which were not included. However, the number of these procedures is small. Moreover, it is important to note that our findings reflect the realities of the selected cities rather than providing a comprehensive overview of the entire state of São Paulo or Brazil as a whole.

## Conclusion

In conclusion, between 2009 and 2019, while the number of hysterectomies performed in the state of São Paulo experienced an increase, there was also a substantial rise in the number of residents in their final year of training. Consequently, despite the increase in total hysterectomies, this period witnessed a reduction in hysterectomy rates per resident during their graduation year, potentially affecting the learning curve within gynecologic surgery.
